# Supplementary material for: End Sequence Analysis Toolkit (ESAT) expands the extractable information from single-cell RNA-seq data
Source: Genome Res. 2016 Oct;26(10):1397–410. doi: 10.1101/gr.207902.116 (PMC5052061; doi:10.1101/gr.207902.116)
Supplement: Supplemental Material [file supp_gr.207902.116_Supp_Materials.tar.gz › ESAT-esat_v0.1/doc/html/classumms_1_1core_1_1annotation_1_1_compound_interval.html]

ESAT: umms.core.annotation.CompoundInterval Class Reference


ESAT


- Main Page
- Related Pages
- Packages
- Classes
- Files

- Class List
- Class Index
- Class Hierarchy
- Class Members

All Classes Namespaces Files Functions Variables Enumerator Pages

- umms
- core
- annotation
- CompoundInterval

Public Member Functions |
List of all members

umms.core.annotation.CompoundInterval Class Reference

Inheritance diagram for umms.core.annotation.CompoundInterval:

[legend]

Collaboration diagram for umms.core.annotation.CompoundInterval:

[legend]

|  |  |
| --- | --- |
| Public Member Functions | |
|  | CompoundInterval () |
|  | |
|  | CompoundInterval (int start, int end) |
|  | |
|  | CompoundInterval (CompoundInterval other) |
|  | |
| int | getStart () |
|  | |
| int | getEnd () |
|  | |
| int | numBlocks () |
|  | |
| int | length () |
|  | |
| int | getSpan () |
|  | |
| int | getCoordinateAtPosition (int positionInInterval) |
|  | |
| int | getPositionAtCoordinate (int coordinateInReference) |
|  | |
| void | setStart (int start) |
|  | |
| void | setEnd (int end) |
|  | |
| void | addInterval (SingleInterval newInterval) |
|  | |
| void | addInterval (int start, int end) |
|  | |
| void | shift (int delta) |
|  | |
| void | moveToCoordinate (int coordinateInReference) |
|  | |
| boolean | containsExactInterval (SingleInterval i) |
|  | |
| boolean | containsInterval (SingleInterval i) |
|  | |
| boolean | contains (CompoundInterval other) |
|  | |
| boolean | overlaps (CompoundInterval other) |
|  | |
| boolean | overlaps (CompoundInterval other, int buffer, boolean ignoreBlocks) |
|  | |
| boolean | overlaps (CompoundInterval other, boolean ignoreBlocks) |
|  | |
| boolean | overlaps (CompoundInterval other, int buffer) |
|  | |
| boolean | overlaps (SingleInterval interval) |
|  | |
| boolean | overlaps (SingleInterval interval, int buffer) |
|  | |
| void | removeInterval (SingleInterval i) |
|  | |
| CompoundInterval | intersect (CompoundInterval other) |
|  | |
| CompoundInterval | union (CompoundInterval other) |
|  | |
| CompoundInterval | complement () |
|  | |
| CompoundInterval | complement (int start, int end) |
|  | |
| CompoundInterval | minus (CompoundInterval other) |
|  | |
| final SortedSet< SingleInterval > | getBlocks () |
|  | |
| boolean | equals (CompoundInterval other) |
|  | |
| int | compareTo (CompoundInterval other) |
|  | |
| String | toString () |
|  | |

## Detailed Description

Author
:   engreitz This class contains multiple non-overlapping intervals on an arbitrary integer coordinate space. Intervals are stored using red-black trees so O(log n) performance is guaranteed.

## Constructor & Destructor Documentation

|  |  |  |  |  |
| --- | --- | --- | --- | --- |
| umms.core.annotation.CompoundInterval.CompoundInterval | ( |  | ) |  |

Here is the caller graph for this function:

|  |  |  |  |
| --- | --- | --- | --- |
| umms.core.annotation.CompoundInterval.CompoundInterval | ( | int | *start*, |
|  |  | int | *end* |
|  | ) |  |  |

Here is the call graph for this function:

|  |  |  |  |  |  |
| --- | --- | --- | --- | --- | --- |
| umms.core.annotation.CompoundInterval.CompoundInterval | ( | CompoundInterval | *other* | ) |  |

Here is the call graph for this function:

## Member Function Documentation

|  |  |  |  |  |  |
| --- | --- | --- | --- | --- | --- |
| void umms.core.annotation.CompoundInterval.addInterval | ( | SingleInterval | *newInterval* | ) |  |

Adds an interval, updating the compound interval to reflect the intersection of its previous state and the new interval.

Author
:   engreitz

Parameters
:   |  |  |
    | --- | --- |
    | newInterval |  |

Here is the caller graph for this function:

|  |  |  |  |
| --- | --- | --- | --- |
| void umms.core.annotation.CompoundInterval.addInterval | ( | int | *start*, |
|  |  | int | *end* |
|  | ) |  |  |

Here is the call graph for this function:

|  |  |  |  |  |  |
| --- | --- | --- | --- | --- | --- |
| int umms.core.annotation.CompoundInterval.compareTo | ( | CompoundInterval | *other* | ) |  |

Here is the call graph for this function:

|  |  |  |  |  |
| --- | --- | --- | --- | --- |
| CompoundInterval umms.core.annotation.CompoundInterval.complement | ( |  | ) |  |

Returns
:   a new CompoundInterval containing the gaps between the blocks in this interval

Here is the call graph for this function:

|  |  |  |  |
| --- | --- | --- | --- |
| CompoundInterval umms.core.annotation.CompoundInterval.complement | ( | int | *start*, |
|  |  | int | *end* |
|  | ) |  |  |

Returns
:   complement of this CompoundInterval using the provided bounds

Here is the call graph for this function:

|  |  |  |  |  |  |
| --- | --- | --- | --- | --- | --- |
| boolean umms.core.annotation.CompoundInterval.contains | ( | CompoundInterval | *other* | ) |  |

Parameters
:   |  |  |
    | --- | --- |
    | other |  |

Returns
:   returns true if all blocks in "other" are contained in blocks of this object

Here is the call graph for this function:

|  |  |  |  |  |  |
| --- | --- | --- | --- | --- | --- |
| boolean umms.core.annotation.CompoundInterval.containsExactInterval | ( | SingleInterval | *i* | ) |  |

Parameters
:   |  |  |
    | --- | --- |
    | i |  |

Returns
:   true if an interval with these exact boundaries is present in the CompoundInterval

Here is the caller graph for this function:

|  |  |  |  |  |  |
| --- | --- | --- | --- | --- | --- |
| boolean umms.core.annotation.CompoundInterval.containsInterval | ( | SingleInterval | *i* | ) |  |

Parameters
:   |  |  |
    | --- | --- |
    | i |  |

Returns
:   true if i is entirely contained within a block (i can be smaller than the block)

Here is the caller graph for this function:

|  |  |  |  |  |  |
| --- | --- | --- | --- | --- | --- |
| boolean umms.core.annotation.CompoundInterval.equals | ( | CompoundInterval | *other* | ) |  |

Parameters
:   |  |  |
    | --- | --- |
    | other |  |

Returns
:   true if all contained intervals are equal

Here is the caller graph for this function:

|  |  |  |  |  |
| --- | --- | --- | --- | --- |
| final SortedSet<SingleInterval> umms.core.annotation.CompoundInterval.getBlocks | ( |  | ) |  |

Returns
:   individual SingleIntervals. should not be modified

Here is the caller graph for this function:

|  |  |  |  |  |  |
| --- | --- | --- | --- | --- | --- |
| int umms.core.annotation.CompoundInterval.getCoordinateAtPosition | ( | int | *positionInInterval* | ) |  |

Parameters
:   |  |  |
    | --- | --- |
    | positionInInterval | 0-based position in the compound interval |

Returns
:   reference coordinate at the given interval position. e.g. for positionInInterval=0, returns getStart()

Here is the call graph for this function:

|  |  |  |  |  |
| --- | --- | --- | --- | --- |
| int umms.core.annotation.CompoundInterval.getEnd | ( |  | ) |  |

Here is the caller graph for this function:

|  |  |  |  |  |  |
| --- | --- | --- | --- | --- | --- |
| int umms.core.annotation.CompoundInterval.getPositionAtCoordinate | ( | int | *coordinateInReference* | ) |  |

Parameters
:   |  |  |
    | --- | --- |
    | coordinateInReference |  |

Returns
:   0-based interval position at given reference coordinate

Here is the call graph for this function:

|  |  |  |  |  |
| --- | --- | --- | --- | --- |
| int umms.core.annotation.CompoundInterval.getSpan | ( |  | ) |  |

Here is the call graph for this function:

|  |  |  |  |  |
| --- | --- | --- | --- | --- |
| int umms.core.annotation.CompoundInterval.getStart | ( |  | ) |  |

Here is the caller graph for this function:

|  |  |  |  |  |  |
| --- | --- | --- | --- | --- | --- |
| CompoundInterval umms.core.annotation.CompoundInterval.intersect | ( | CompoundInterval | *other* | ) |  |

Parameters
:   |  |  |
    | --- | --- |
    | other |  |

Returns
:   new CompoundInterval containing intersections of the blocks of the two objects

Here is the call graph for this function:

Here is the caller graph for this function:

|  |  |  |  |  |
| --- | --- | --- | --- | --- |
| int umms.core.annotation.CompoundInterval.length | ( |  | ) |  |

Here is the caller graph for this function:

|  |  |  |  |  |  |
| --- | --- | --- | --- | --- | --- |
| CompoundInterval umms.core.annotation.CompoundInterval.minus | ( | CompoundInterval | *other* | ) |  |

Here is the call graph for this function:

|  |  |  |  |  |  |
| --- | --- | --- | --- | --- | --- |
| void umms.core.annotation.CompoundInterval.moveToCoordinate | ( | int | *coordinateInReference* | ) |  |

Move an annotation, preserving the relationships between its blocks, to a new coordinate.

Parameters
:   |  |  |
    | --- | --- |
    | coordinateInReference |  |

Here is the call graph for this function:

|  |  |  |  |  |
| --- | --- | --- | --- | --- |
| int umms.core.annotation.CompoundInterval.numBlocks | ( |  | ) |  |

Here is the caller graph for this function:

|  |  |  |  |  |  |
| --- | --- | --- | --- | --- | --- |
| boolean umms.core.annotation.CompoundInterval.overlaps | ( | CompoundInterval | *other* | ) |  |

Parameters
:   |  |  |
    | --- | --- |
    | other |  |

Returns
:   returns true if any block in "other" overlaps with any block of this object

Here is the caller graph for this function:

|  |  |  |  |
| --- | --- | --- | --- |
| boolean umms.core.annotation.CompoundInterval.overlaps | ( | CompoundInterval | *other*, |
|  |  | int | *buffer*, |
|  |  | boolean | *ignoreBlocks* |
|  | ) |  |  |

Parameters
:   |  |  |
    | --- | --- |
    | other |  |
    | ignoreBlocks | if true, then overlap will consider only the start and end boundaries, ignoring blocks |

Returns
:   true if the two intervals overlap

Here is the call graph for this function:

|  |  |  |  |
| --- | --- | --- | --- |
| boolean umms.core.annotation.CompoundInterval.overlaps | ( | CompoundInterval | *other*, |
|  |  | boolean | *ignoreBlocks* |
|  | ) |  |  |

Parameters
:   |  |  |
    | --- | --- |
    | other |  |
    | ignoreBlocks |  |

Returns
:   true if the boundaries of the intervals overlap

Here is the call graph for this function:

|  |  |  |  |
| --- | --- | --- | --- |
| boolean umms.core.annotation.CompoundInterval.overlaps | ( | CompoundInterval | *other*, |
|  |  | int | *buffer* |
|  | ) |  |  |

Parameters
:   |  |  |
    | --- | --- |
    | other |  |
    | buffer |  |

Returns
:   true if any of the blocks come within the buffer distance of each other

Here is the call graph for this function:

|  |  |  |  |  |  |
| --- | --- | --- | --- | --- | --- |
| boolean umms.core.annotation.CompoundInterval.overlaps | ( | SingleInterval | *interval* | ) |  |

Parameters
:   |  |  |
    | --- | --- |
    | interval |  |

Returns
:   returns true if "interval" overlaps with any block of this object

Here is the call graph for this function:

|  |  |  |  |
| --- | --- | --- | --- |
| boolean umms.core.annotation.CompoundInterval.overlaps | ( | SingleInterval | *interval*, |
|  |  | int | *buffer* |
|  | ) |  |  |

Parameters
:   |  |  |
    | --- | --- |
    | interval |  |
    | buffer |  |

Returns
:   true if any block comes within the buffer distance from the given interval

Here is the call graph for this function:

|  |  |  |  |  |  |
| --- | --- | --- | --- | --- | --- |
| void umms.core.annotation.CompoundInterval.removeInterval | ( | SingleInterval | *i* | ) |  |

Remove an interval. Throws exception if the interval does not exist - so check first.

Parameters
:   |  |  |
    | --- | --- |
    | i |  |

Here is the call graph for this function:

|  |  |  |  |  |  |
| --- | --- | --- | --- | --- | --- |
| void umms.core.annotation.CompoundInterval.setEnd | ( | int | *end* | ) |  |

Extends or trims the end coordinate. If trimming, removes blocks that fall after the new end coordinate. If expanding, extends the last block to the new end coordinate.

Parameters
:   |  |  |
    | --- | --- |
    | end | new end coordinate |

Here is the call graph for this function:

|  |  |  |  |  |  |
| --- | --- | --- | --- | --- | --- |
| void umms.core.annotation.CompoundInterval.setStart | ( | int | *start* | ) |  |

Extends or trims the start coordinate. If trimming, removes any blocks that fall before the new start position and truncates the block that overlaps the start position, if it exists. If expanding, extends the first block to the new start position.

Parameters
:   |  |  |
    | --- | --- |
    | start | new start coordinate |

Here is the call graph for this function:

|  |  |  |  |  |  |
| --- | --- | --- | --- | --- | --- |
| void umms.core.annotation.CompoundInterval.shift | ( | int | *delta* | ) |  |

Shift all blocks in the compound interval by a given delta

Parameters
:   |  |  |
    | --- | --- |
    | delta |  |

Here is the caller graph for this function:

|  |  |  |  |  |
| --- | --- | --- | --- | --- |
| String umms.core.annotation.CompoundInterval.toString | ( |  | ) |  |

|  |  |  |  |  |  |
| --- | --- | --- | --- | --- | --- |
| CompoundInterval umms.core.annotation.CompoundInterval.union | ( | CompoundInterval | *other* | ) |  |

Parameters
:   |  |  |
    | --- | --- |
    | other |  |

Returns
:   new CompoundInterval containing the (blocked) union of the two objects

Here is the call graph for this function:

---

The documentation for this class was generated from the following file:

- java/umms/core/annotation/CompoundInterval.java


---

Generated on Fri Aug 15 2014 15:11:08 for ESAT by  

 1.8.7
